# Exploration and identification of six novel ferroptosis-related hub genes as potential gene signatures for peripheral nerve injury

**DOI:** 10.3389/fgene.2023.1156467

**Published:** 2023-04-06

**Authors:** Yifei Zhang, Chun Chen, Dawei Li, Penghui Chen, Lei Hang, Jun Yang, Jin Xie

**Affiliations:** ^1^ Department of Otorhinolaryngology-Head and Neck Surgery, Xinhua Hospital, Shanghai Jiao Tong University School of Medicine, Shanghai, China; ^2^ Ear Institute, Shanghai Jiao Tong University School of Medicine, Shanghai, China; ^3^ Shanghai Key Laboratory of Translational Medicine on Ear and Nose Diseases, Shanghai, China; ^4^ Business School, Tianhua College, Shanghai Normal University, Shanghai, China

**Keywords:** peripheral nerve regeneration, ferroptosis, biomarker, bioinformatics, hub gene, peripheral nerve injury

## Abstract

Specific biomarkers of ferroptosis after peripheral nerve injury (PNI) are still under debate. In this study, 52 differentially expressed ferroptosis-related genes (DE-FRGs) were retrieved from publicly accessible sequencing data of intact and injured samples of rats with sciatic nerve crush injury. Functional enrichment analyses revealed that adipogenesis, mitochondrial gene sets, and pathways of MAPK, p53, and CD28 family were predominantly engaged in ferroptosis after PNI. Next, Cdkn1a, Cdh1, Hif1a, Hmox1, Nfe2l2, and Tgfb1 were investigated as new ferroptosis-associated hub genes after PNI. Subsequently, clustering correlation heatmap shows six hub genes are linked to mitochondria. The immunofluorescence assay at 0, 1, 4, 7, and 14 days indicated the temporal expression patterns of Tgfb1, Hmox1, and Hif1a after PNI were consistent with ferroptosis validated by PI and ROS staining, while Cdh1, Cdkn1a, and Nfe2l2 were the opposite. In summary, this study identified six hub genes as possible ferroptosis-related biomarkers for PNI, which may offer therapeutic targets for peripheral nerve regeneration and provide a therapeutic window for ferroptosis.

## Introduction

Peripheral nerve injury (PNI) is often caused by trauma-related injuries (Campbell, 2008). Each year, many patients suffer from PNI, making it a serious clinical and public health issue (Noble et al., 1998; Robinson, 2000). Approximately one-third of all PNI are linked to insufficient nerve healing and poor functional outcomes (Atkins et al., 2006). However, existing therapies have not entirely achieved anticipated outcomes (Faroni et al., 2015). Therefore, it is essential to increase our understanding of the cellular and molecular mechanisms and to develop novel treatment strategies based on a comprehensive understanding of the biological processes underlying PNI and regeneration.

PNI pathophysiology is complex. Due to Schwann cells (SCs) plasticity, the peripheral nervous system (PNS) regenerates better than the central nervous system (CNS) (Nocera and Jacob, 2020). PNI reprograms SCs to a repair phenotype. Repair SCs remove axons and myelin debris, sustain neuronal survival, provide trophic factors, and regenerate axons (Jessen and Mirsky, 2016). So, SCs are crucial to PNI healing. Yet, some scientists have found that SCs decrease dramatically in late-stage PNI compared to early-stage (Siironen et al., 1994; Li et al., 1998; Jonsson et al., 2013). Chronically denervated stumps’ decreased regeneration may be due to a progressive decrease in SCs or a weakening of the SCs’ repair phenotype (Jessen and Mirsky, 2019). Why are SCs falling? Cell death may cause this. Ferroptotic cell death has been linked.

Dixon et al. coined the term “ferroptosis” to characterize iron-dependent, lipid peroxide-induced programmed cell death (Dixon et al., 2012). Ferroptosis lacks apoptotic features yet has fewer mitochondria and shrunken mitochondria (Stockwell et al., 2017). Ferroptosis occurs when the execution mechanisms outnumber the defensive systems, unlike other programmed cell deaths with key death executor proteins (Galluzzi et al., 2018). Mitochondria are one of the main sites where ferroptosis occurs, so they are important for ferroptosis (Gao et al., 2019). Recent studies have shown that ferroptosis occurs after PNI (Guo et al., 2021; Gao et al., 2022). However, the specific ferroptosis biomarkers after PNI are still inconclusive, implying that more research into ferroptosis-related genes is required.

In this study, we aimed to identify specific ferroptosis-associated biomarkers after PNI. A dataset was acquired from the Gene Expression Omnibus (GEO) and bioinformatics analyses were performed to select differentially expressed ferroptosis-related genes (DE-FRGs). After functional enrichment analysis and protein-protein interaction (PPI) network construction, six ferroptosis-related hub genes were identified, which includes Cdkn1a, Cdh1, Hif1a, Hmox1, Nfe2l2, and Tgfb1. Finally, mitochondrial correlation analysis, PI staining, ROS staining, and immunofluorescence assay was carried out to verify our theory.

## Materials and methods

### Data acquisition

Multiple gene expression data from high-throughput RNA-sequencing were downloaded from the Gene Expression Omnibus (GEO) database and Sequence Read Archive (SRA) database with series numbers GSE177037, GSE162548, and SRP113121. All three datasets contain sequencing data of rat sciatic nerves at different time points after crush injury. GSE177037 was used to pinpoint the ferroptosis-related signature, while GSE162548 and SRP113121 were used to confirm the accuracy of the findings. Data sets of ferroptosis related genes (FRGs), including marker, driver, and suppressor, were retrieved from FerrDb Database. After removing the duplicated genes, 487 FRGs validated previously were selected for subsequent research.

### Differential expression analysis

Differential expression analysis permits the comparison of the relative expression of all sequenced genes between two distinct samples in the dataset, thereby identifying statistically significant genes. Briefly, the database for annotation, visualization, and integrated discovery (DAVID) was used to convert Entrez gene ID in the raw count to official gene symbol, then “DESeq2” package in the R program (version 4.1.3) was utilized to calibrate the original data and calculate the differentially expressed genes (DEGs) in sciatic nerves 3, 5, and 7 days after crush injury and in uninjured sciatic nerves. The protein coding RNA that meets the defined criteria, |log2(FC)|>1 and adjusted *p*-value<0.05, were considered as DEGs. The DEGs were displayed in advanced volcano plots based on the “ggplot2”, “OmicStudioClassic” and “OmicStudioKits” package. The intersecting genes between 3 days-DEGs, 5 days-DEGs, 7 days-DEGs and FRGs were defined as the differentially expressed ferroptosis-related genes (DE-FRGs). The number of DE-FRGs was shown in a Venn diagram using the “VennDiagram” package. A total of 52 genes interacted with each of these segments and a total of 108 genes had at least one intersection with FRGs. To visualize the expression of these overlapping genes. The “stats” package was used for cluster calculation and Pearson’s correlation analysis, and “pheatmap” package was used to draw clustering heatmap and clustering correlation heatmap.

### Function enrichment analysis

Gene Set Enrichment Analysis (GSEA) was used to explore the expression of injured and non-injured sciatic nerve samples in the preset gene set. The expression data of all genes were uploaded and used for statistics and display with “clusterProfiler” and “enrichplot” package, respectively. The preset gene set was obtained from Molecular Signatures Database (MSigDB) database. Gene sets satisfied the adjusted *p*-value < 0.05, False Discovery Rate (FDR) < 0.25, and |Normalized Enrichment Scores (NES)| >1 were shown. *p* values are corrected by Benjamini–Hochberg method.

Biological Process (BP), Cellular Component (CC) and Molecular Function (MF) from Gene Ontology (GO) database and Kyoto Encyclopedia of Genes and Genomes (KEGG) pathway enrichment analyses using the “ClusterProfiler” package were performed to obtain insights into the potential functions of the 52 intersection genes. Enrichment analyses from the REACTOME and WikiPathways databases were used to complement the above results. The top 20 results were shown in the enrichment scatter plots.

### Protein-protein interaction network analysis

Protein-protein interaction (PPI) networks consist of the interaction relationships between proteins. Systematic analysis of the interaction relationships of a large number of proteins in the organism enables us to study the working principles of specific proteins, investigate key biological signals in certain pathophysiological processes and key mechanisms in the metabolism of energy substances, and comprehend the functional connections between proteins. Briefly, the STRING database was employed to analyze the interactions of 108 genes that had at least one intersection with FRGs as described above. After removing disconnected nodes in the network, data is sent to Cytoscape software v3.9.1 for further analysis. The top six genes in PPI network were selected as hub genes, which were comprehensively analyzed referred to the Betweenness Centralities (BC), Closeness Centralities (CC) and Degree Centralities (DC) by using cytoNCA plugin.

### Correlation analysis of hub genes and MRGs

The most up-to-date version of the information was taken from the Integrated Mitochondrial Protein Index (IMPI) database. Selected genes have been validated by prior studies and were predicted to be mitochondrial related genes (MRGs) by experiments/articles. A total of 2008 MRGs were screened for further analysis. The intersecting genes of the 3 days DEGs, 5 days DEGs, 7 days DEGs and MRGs were defined as the differentially expressed mitochondrial-related genes (DE-MRGs). Pearson’s correlation analysis between the hub genes and DE-MRGs was performed utilizing the “stats” package. All results were displayed in a clustering correlation heatmap.

### Animal

Twenty adult male Sprague-Dawley (SD) rats, weighing 110–130 g, were acquired from Shanghai Slac Laboratory Animal Co., Ltd. (Shanghai, China). Rats were housed in colony rooms with monitored humidity and temperature and were maintained in groups of five, with a 12-h light/dark cycle. All animal experiments are conducted in compliance with the rules established by the Experimental Animals Ethics Committee at Xinhua Hospital Affiliated to Shanghai Jiao Tong University School of Medicine.

### Peripheral nerve injury model

The 20 rats were randomly divided into five groups (designated as 0, 1, 4, 7, and 14 days; n = 4 rats/group). After anesthetizing the rats with a mixture of anaesthetics which include 65 mg/kg Zoletil 50 (Virbac, Carros, France) and 10 mg/kg Serazine hydrochloride (HUAMU, Jilin, China), rat right sciatic nerves were dissected out. At a point roughly 5 mm proximal to the bifurcation of the tibial and fibular nerves, the nerve was clamped three times with forceps for 10 s each, followed by layer-by-layer suturing. Rats were sacrificed by cervical dislocation at 0, 1, 4, 7, and 14 days after surgery. A 10 mm long sciatic nerve stump at the crush site was collected for subsequent experiments. Notably, the sciatic nerves in the 0-day group were collected directly without crush injury and served as a control group.

### PI staining

Samples of fresh sciatic nerve were fixed in 4% paraformaldehyde (G1101, Servicebio, Wuhan, China) for 24 h, dehydrated *via* a gradient of ethanol, embedded in paraffin, and then sectioned using a paraffin microtome (RM 2016, Leica, Wetzlar, Germany). The tissue sections were directly mounted onto glass slides for staining and storage. Then, the samples were incubated with Propidium Iodide (PI, G1021, Servicebio, Wuhan, China) in the dark for 10 min and stained with diamidino-2-phenylindole (DAPI, G1012, Servicebio, Wuhan, China) for 10 min before observation.

### Reactive oxygen species (ROS) experiment

Fresh samples were embedded with OCT Compound (4,583, Sakura Finetek, Nagano, Japan) and sectioned with a Cryostat (Cryostar NX50, Thermo Fisher Scientific, Waltham, MA, United States). After rewarming at room temperature, the sections were incubated with ROS indicator dihydroethidium (DHE, D7008, SIGMA, St. Louis, MO, United States of America) at 37°C for 30 min in the dark, and then the nuclei were counterstained with DAPI for 10 min. Finally, inverted fluorescence microscope (IX73, Olympus, Tokyo, Japan) was used to observe.

### Immunofluorescence

Paraffin sections of the sciatic nerve were prepared using the same method as before. After dewaxing, the sections were blocked with 3% BSA (GC305006, Servicebio, Wuhan, China) in PBS (G4202, Servicebio, Wuhan, China) for 30 min. The tissue sections were incubated sequentially with primary and secondary antibodies. The sections were subsequently incubated for 10 min using DAPI and protected from light. Then, antifade mounting medium (P0126, Beyotime, Shanghai, China) was added and observed using fluorescence microscope (Eclipse C1, Nikon, Tokyo, Japan). Details can be found in Table 1.

**TABLE 1:** List of antibodies used in this study.

Antibody	Source	Catalog	Species	Dilution	Incubation time	Temperature
Anti-Cdh1	Servicebio, Wuhan, China	GB12082	Mouse	1:500	Overnight	4 °C
Anti-Cdkn1a	Abclonal, Wuhan, China	A1483	Rabbit	1:100	Overnight	4 °C
Anti-Hif1a	Abmart, Shanghai, China	TA1009	Rabbit	1:200	Overnight	4 °C
Anti-Hmox1	Servicebio, Wuhan, China	GB12104	Mouse	1:500	Overnight	4 °C
Anti-Nfe2l2	Abclonal, Wuhan, China	A0674	Rabbit	1:100	Overnight	4 °C
Anti-Tgfb1	Abclonal, Wuhan, China	A21245	Rabbit	1:100	Overnight	4 °C
Anti-S100β	Servicebio, Wuhan, China	GB113884	Rabbit	1:500	Overnight	4 °C
Anti-S100β	Servicebio, Wuhan, China	GB12360	Mouse	1:500	Overnight	4 °C
FITC Goat Anti-Rabbit	Servicebio, Wuhan, China	GB22303	Goat	1:100	1 h	Room Temperature
Cy3 Goat Anti-Rabbit	Servicebio, Wuhan, China	GB21303	Goat	1:300	1 h	Room Temperature
FITC Goat Anti-Mouse	Servicebio, Wuhan, China	GB22301	Goat	1:100	1 h	Room Temperature
Cy3 Goat Anti-Mouse	Servicebio, Wuhan, China	GB21301	Goat	1:300	1 h	Room Temperature

### Statistical analysis

The original data in the dataset followed a negative binomial distribution, hence hypothesis testing with parametric tests, which led to differential expression analysis of the various samples. Before differential expression analysis, each group’s data is first normalized. Afterwards, the standardized matrices were statistically evaluated using the “DESeq2” package (Love et al., 2014), and then DEGs were identified using the criteria |log2(FC)| >1 and an adjusted *p*-value < 0.05. Details of the statistics in other experiments have been elucidated in the above methods.

Whenever comparing more than two groups, one-way analysis of variance (ANOVA) was employed, whereas two-tailed Student's t-tests were utilized to analyze comparisons between two groups. At least three separate experiments were used to collect all of the data. All statistical analyses and visualizations were performed in the R program (version 4.1.3). If not otherwise specified, a *p*-value of less than 0.05 was considered statistically significant.

## Results

### Identification of DE-FRGs in sciatic nerve after crush injury

**FIGURE 1:**
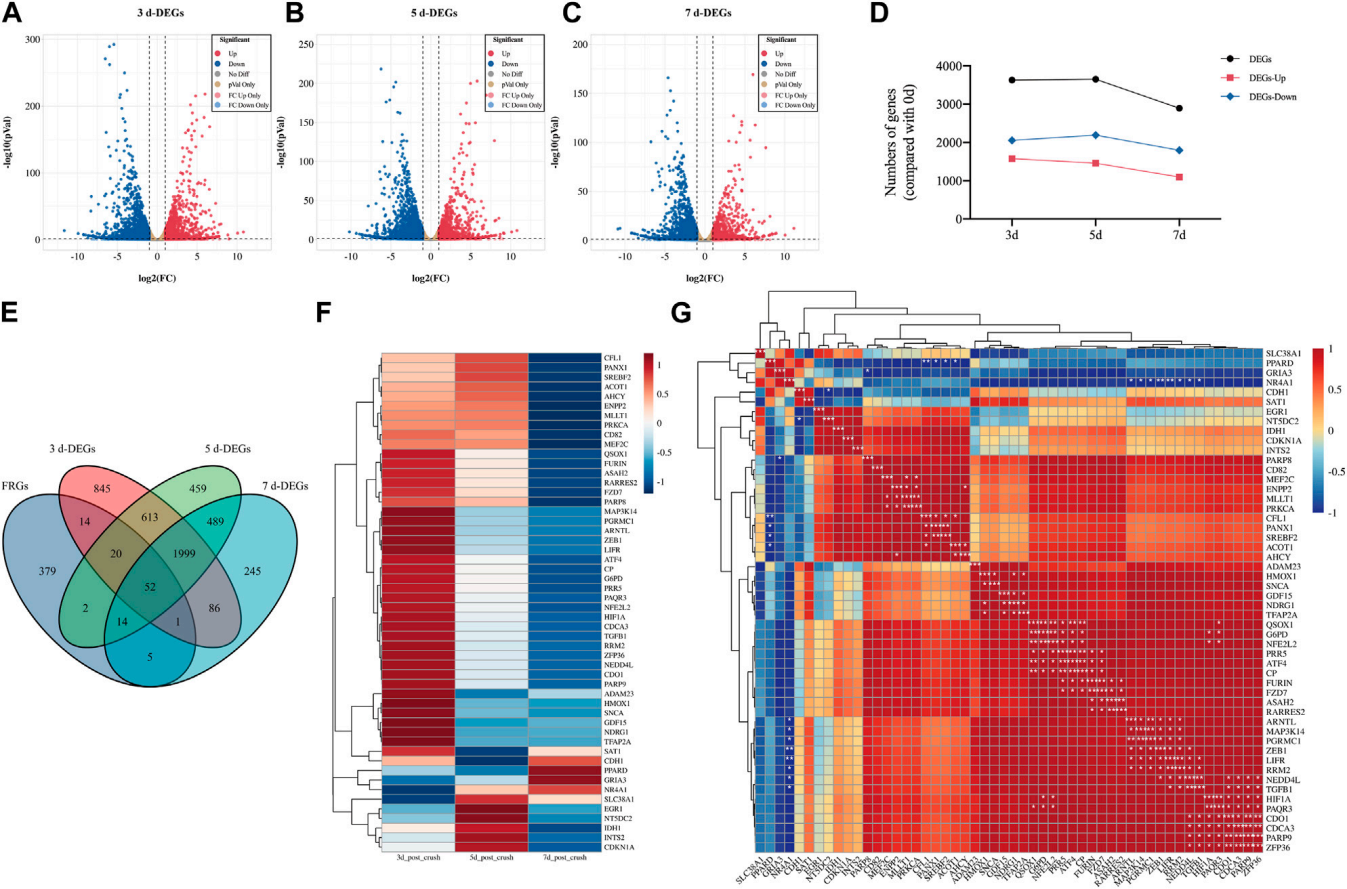
Identification of DE-FRGs in sciatic nerve after crush injury. **(A–C)** Volcano plots of differentially expressed genes (DEGs) of 3-day group, 5-day group, and 7-day group after sciatic nerve injury compared to 0-day group (uninjured, control). Data was obtained from GSE177037. Red dots represent significantly upregulated genes and blue dots represent significantly downregulated genes with an adjusted *p*-value <0.05 and |log2FC|>1. **(D)** A line chart of the number of genes of DEGs, upregulated DEGs and downregulated DEGs over time. **(E)** Venn diagram shows the DE-FRGs. **(F)** The clustering heatmap displays the relative expression extent of 52 DE-FRGs at 3-day group, 5-day group, and 7-day group post crush injury compared to 0-day group. The level of expression increases with increasing redness and decreases with increasing blueness. The clustering results are shown on the left of the image. **(G)** The clustering correlation heatmap represents significant correlations among 52 DE-FRGs. The clustering results are shown at the left and top of the image. The color bars indicate the degree of correlation between genes. A darker red indicates a more positive correlation, while a darker blue indicates a more negative correlation. * indicates significance level, *p < 0.05, **p < 0.01, ***p < 0.001.

GSE177037, a high-throughput sequencing dataset obtained from the GEO database, was selected for further investigations on DEGs in sciatic nerve following crush injury. The results of DEGs at 3, 5, and 7 days after sciatic nerve injury are shown with volcanic plots. (Figures 1A–C). In the 3-day group, there were 3,630 DEGs, including 2052 downregulated genes and 1,578 upregulated genes. In the 5-day group, there were 3,648 DEGs, including 2,191 downregulated genes and 1,457 upregulated genes. The 7-day group had 2,891 DEGs, 1,096 of which were upregulated and 1795 of which were downregulated. It can be found that the number of DEGs gradually decreased with time, and the number of downregulated genes was slightly more than that of upregulated genes. (Figure 1D).

Then, we obtained information from the FerrDb database, a regularly updated database that lists regulators of ferroptosis and associations between ferroptosis and diseases. We selected gene regulators including driver, suppressor, and marker. After removing duplication, a total of 487 genes were obtained as FRGs for subsequent analysis. Using a Venn diagram (Figure 1E), we found that 52 genes were co-expressed in the four groups of FRGs, 3-day group, 5-day group, and 7-day group, and 108 genes met the condition that FRG had an intersection with at least one of the other three groups. Thus, 52 genes represented DE-FRGs that were consistently expressed at all three time periods, while 108 genes represented DE-FRGs that were present at least at one time point.

The clustering heatmap of 52 DE-FRGs was displayed in Figure 1F. After sciatic nerve damage, the expression of the majority of genes in 52 typical DE-FRGs was initially dramatically upregulated and subsequently progressively downregulated. The clustering correlation heatmap of 52 DE-FRGs was shown in Figure 1G. The most of the 52 DE-FRGs were found to be positively linked with other genes. Many of them are significantly correlated.

### Gene set enrichment analysis (GSEA)

**FIGURE 2:**
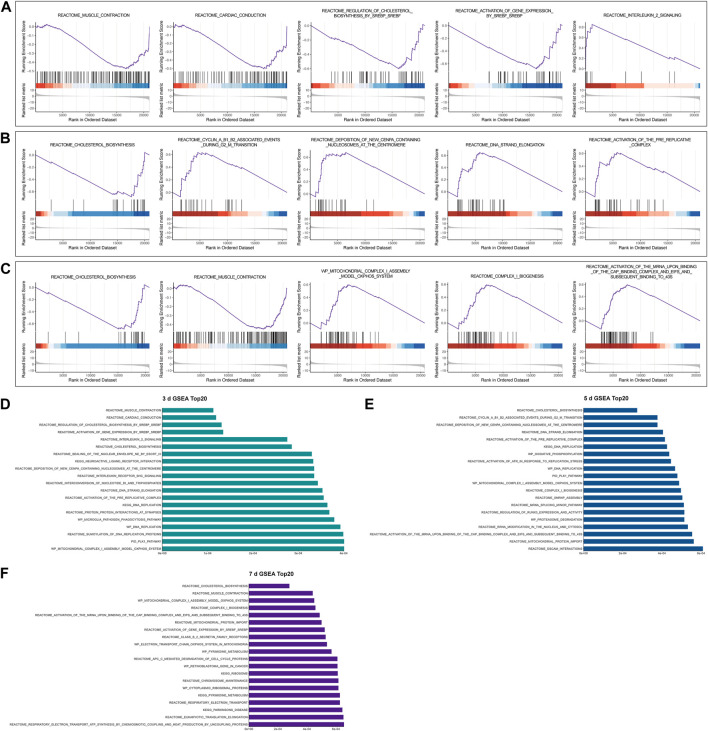
Representative results of gene set enrichment analysis (GSEA). **(A-C)** GSEA at 3 days, 5 days, and 7 days following sciatic nerve crush injury compared to control, respectively. All gene sets satisfy adjusted *p* < 0.05, FDR < 0.25, |NES| >1. **(D-F)** Top 20 results of GSEA in 3-day group, 5-day group, and 7-day group, respectively.

GSEA was used to investigate whether gene sets would be potentially crucial in samples at various stages following nerve damage. According to our analysis of relative fold changes of gene expression data from undamaged nerves and 3, 5, and 7 days after nerve injury, Muscle Contraction, Cardiac Conduction, Regulation of Cholesterol Biosynthesis by SREBP (SREBF), Activation of Gene Expression by SREBP (SREBF), and Interleukin 2 Signaling Pathway were considerably enriched in the 3-day group (Figure 2A). The gene sets Cholesterol Biosynthesis, Cyclin A B1 B2 Associated Events During G2-M Transition, Deposition of New CENPA Containing Nucleosomes Centromere, DNA Strand Elongation, and Activation of the Pre-Replicative Complex were drastically enriched in the 5-day group (Figure 2B), whereas gene sets Cholesterol Biosynthesis, Muscle Contraction, Mitochondrial Complex I Assembly Model OXPHOS System, Complex I Biogenesis, Activation of The mRNA Upon Binding of The Cap Binding Complex And EIFS And Subsequent Binding to 43s was significantly enriched in the 7-day group (Figure 2C).

We also demonstrate the top 20 GSEA results in the 3-day group, 5-day group, and 7-day group in order of significance (Figures 2D–F). Interestingly, we found that the lipid anabolic pathway including Cholesterol Biosynthesis, and Activation of Gene Expression by SREBP (SREBF) was enriched and downregulated multiple times. This suggests to us that the altered expression of these gene sets may be related to the downregulation of myelin genes and the reprogramming of SCs in the first few days after PNI (Jessen and Mirsky, 2016). In addition, the mitochondria-associated genes set including Mitochondrial Protein Import, Mitochondrial Complex I Assembly Model OXPHOS System were also enriched several times, suggesting that those may play an important role after injury.

### Functional enrichment analysis of 52 DE-FRGs

GO, KEGG, Reactome, and WikiPathways databases were used in biological functions and pathway enrichment analyses in an effort to identify any gene sets of high significance among 52 DE-FRGs. Enrichment scatter plots displayed the leading twenty findings.

**FIGURE 3:**
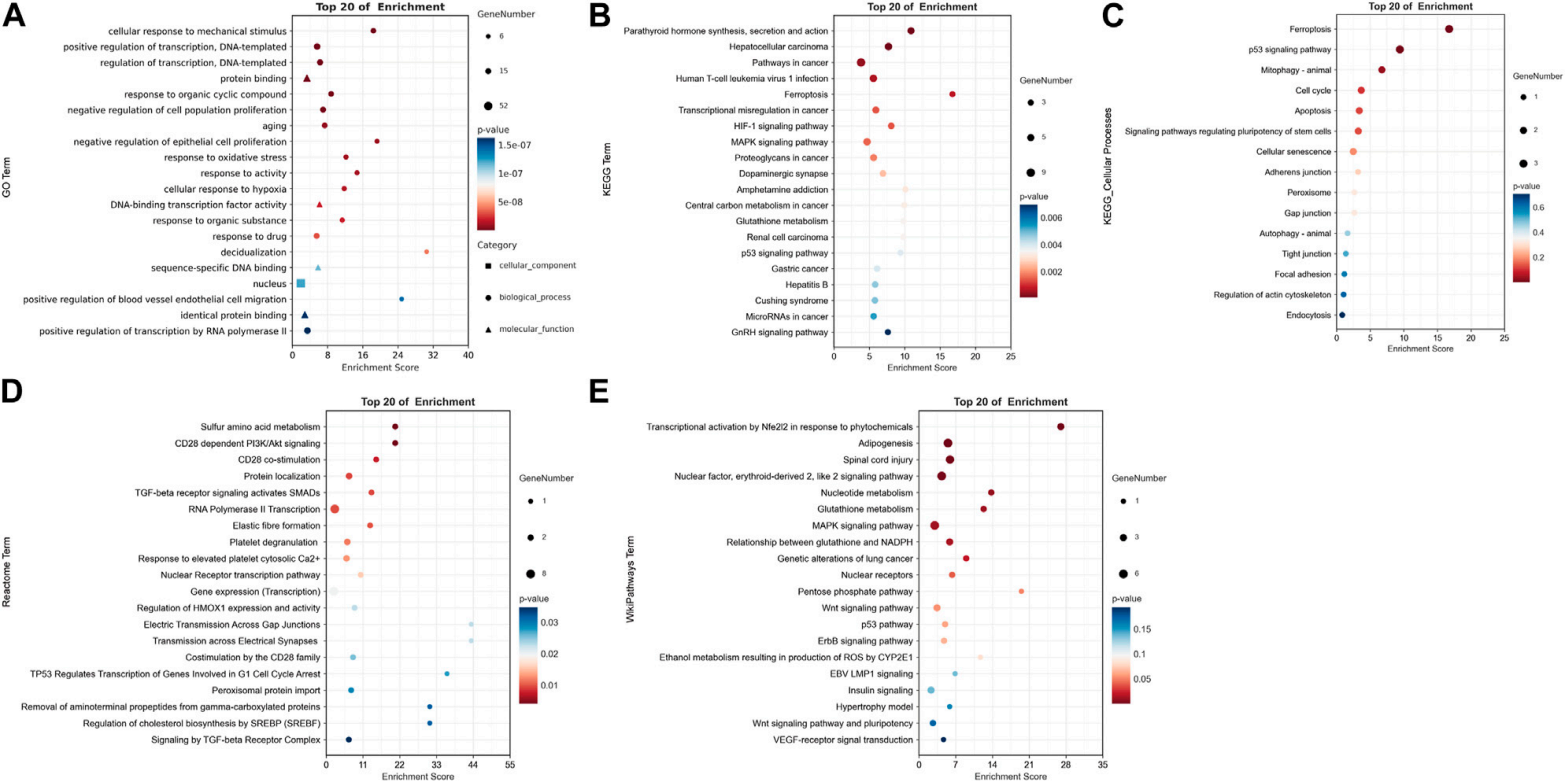
Representative results of functional enrichment analysis enriched by 52 DE-FRGs. **(A)** GO enrichment analysis. **(B)** KEGG pathway enrichment analysis. **(C)** Cellular processes in KEGG pathway enrichment analysis. **(D)** Reactome pathway enrichment analysis. **(E)** WikiPathways enrichment analysis. **A** In GO term, bubbles with square, round, and triangle shapes stand for cellular component, biological process, and molecular function, respectively. **(A–E)** The size of the bubble shows the number of enriched genes. Red indicates smaller *p*-values and blue indicates larger *p*-values. All results satisfy *p* < 0.05.

Based on GO analysis, the results of subclassification biological processes are interesting (Figure 3A). The 52 DE-FRGs were dramatically enriched in related biological processes including cellular response to mechanical stimulus, negative regulation of cell population proliferation, negative regulation of epithelial cell proliferation, positive regulation of blood vessel endothelial cell migration, and positive regulation of transcription by RNA polymerase II. This suggests that multiple cell proliferation and migration following crush injury in our peripheral nerves may be related to ferroptosis. In addition, we found that oxidation-related pathways including response to oxidative stress, and cellular response to hypoxia were also significantly enriched in. Depending on the findings of the KEGG pathway enrichment analysis, ferroptosis may be strongly correlated with the HIF-1 signaling pathway, MAPK signaling pathway, p53 signaling pathway, and GnRH signaling pathway (Figure 3B). Ferroptosis was enriched first in its subclassified cellular processes (Figure 3C), demonstrating the validity of this enrichment finding.

In addition, the metabolic pathway database Reactome and the Wikipathways database were utilized to supplement the functional enrichment results (Figures 3D,E). In the enrichment analysis of the Reactome database, peroxisomal protein import and regulation of cholesterol biosynthesis by SREBP (SREBF) were considerably enriched, which echoed the GSEA results. Furthermore, there was a notable enrichment of pathways associated with the CD28 molecular family, including the PI3K/Akt signaling pathway, CD28 co-stimulation, and costimulation by the CD28 family. As shown by WikiPathways enrichment analysis, adipogenesis, the p53 pathway, the ErbB signaling pathway, the Wnt signaling pathway, and the MAPK signaling pathway may all be involved in ferroptosis.

### Protein-protein interaction network construction and hub genes selection

**FIGURE 4:**
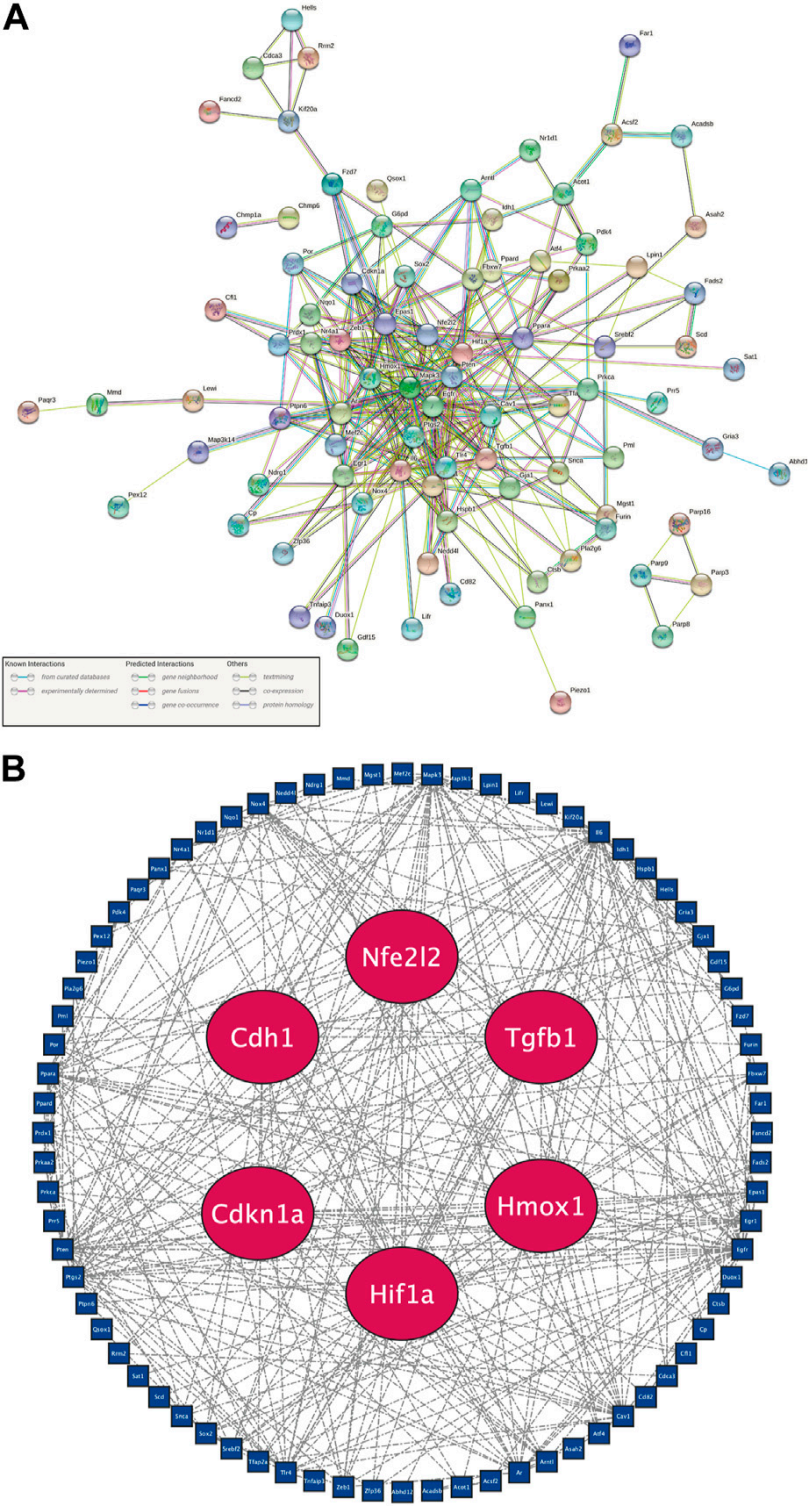
Recognition of six hub genes from 108 candidate DE-FRGs. **(A)** PPI network plot downloaded from the STRING database. Genes were represented as nodes, and relationships between them by edges. A node’s relationships with other genes are more extensive the more edges it is related to. **(B)** PPI network plot of candidate DE-FRGs after deleting unconnected nodes. Hub genes are shown by red nodes, and other genes are shown by blue nodes.

In order to incorporate adequate candidate genes, 108 DE-FRGs were selected to screen for hub genes. The STRING online database provided the 105 nodes and 327 edges of the PPI network (Figure 4A). Three of the 108 genes were unidentified, and seventeen were unrelated to other molecules and did not create a molecular network. The default threshold for the network (Interaction Score >0.4) was used. Nodes represented genes, while edges showed how the genes interacted between themselves.

After further elimination of independent DE-FRGs, Cytoscape software was employed to do further analysis (Figure 4B). The sensitivity and specificity of hub genes must be taken into account in order to choose representative hub genes. Gene differential expression is more likely to be found at various time periods and is more sensitive the longer it persists following peripheral nerve damage. The specificity increases with the score comprising BC, CC, and DC. Consequently, Cdkn1a, Cdh1, Hif1a, Hmox1, Nfe2l2, and Tgfb1 were deemed to be hub genes based on the screening standards of sensitivity, specificity, statistical significance, and novelty in all nodes of the PPI network studied here.

### Correlation between hub genes and mitochondria

Ferroptosis is a kind of cell death that is reliant on the presence of iron and results in the lipid peroxidation of the cell membrane. Mitochondria are crucial areas in which ferroptosis prerequisites and ferroptosis defensive mechanisms occur (Gao et al., 2019). As a result, mitochondria play a significant role in the process of ferroptosis. Non-etheless, the correlation between the hub genes that we have chosen, and mitochondria is still unknown.

**FIGURE 5:**
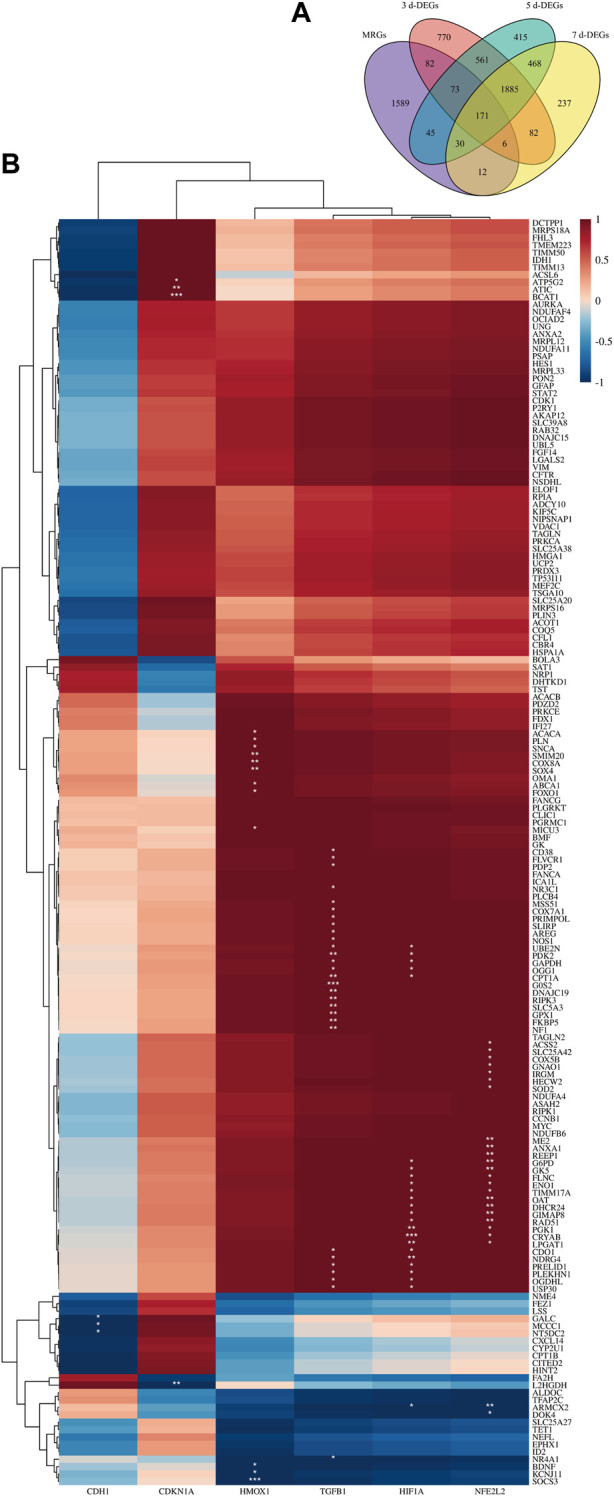
Correlation between hub genes and mitochondria. **(A)** Venn diagram shows the DE-MRGs. **(B)** The clustering correlation heatmap revealed the correlation between 171 DE-MRGs and 6 hub genes. The clustering results are displayed to the image’s left and top. The color bars represent the correlation between genes. A deeper red indicates a stronger positive correlation, while a deeper blue indicates a stronger negative one. * denotes significance level, *p < 0.05, **p < 0.01, ***p < 0.001.

To explain this, DE-MRGs were initially found. First, we retrieved the most recent data from the IMPI database, which included genes encoding mitochondria-related proteins that have been described in the published research and genes that have not been reported but are projected to be closely connected with mitochondria. In all, 2008 MRGs were incorporated into our research after compilation. Venn diagrams illustrated the cross-linking of MRGs and DEGs in the 3-day, 5-day, and 7-day groups (Figure 5A). 171 MRGs were consistently differently expressed across all groups; hence, they were chosen as DE-MRGs.

Subsequently, clustering correlation heatmap analysis (Figure 5B) was performed. The six hub genes can be roughly separated into three clusters based on the clustering results and various expression patterns of linked genes. The first cluster includes Cdh1, the second includes Cdkn1a, and the third cluster includes Hmox1, Tgfb1, Hif1a, and Nfe2l2. We next investigated the connections between the six hub genes and 171 DE-MRGs. The majority of DE-MRGs were strongly positively linked with Hmox1, Hif1a, Nfe2l2, and Tgfb1. Cdkn1a was considerably found to be relevant with a small proportion of DE-MRGs, while Cdh1 was strongly negatively correlated with a small number of DE-MRGs. These results side by side corroborate that our selected hub genes are closely related to ferroptosis.

### Expression validation and temporal alteration identification of hub genes

**FIGURE 6:**
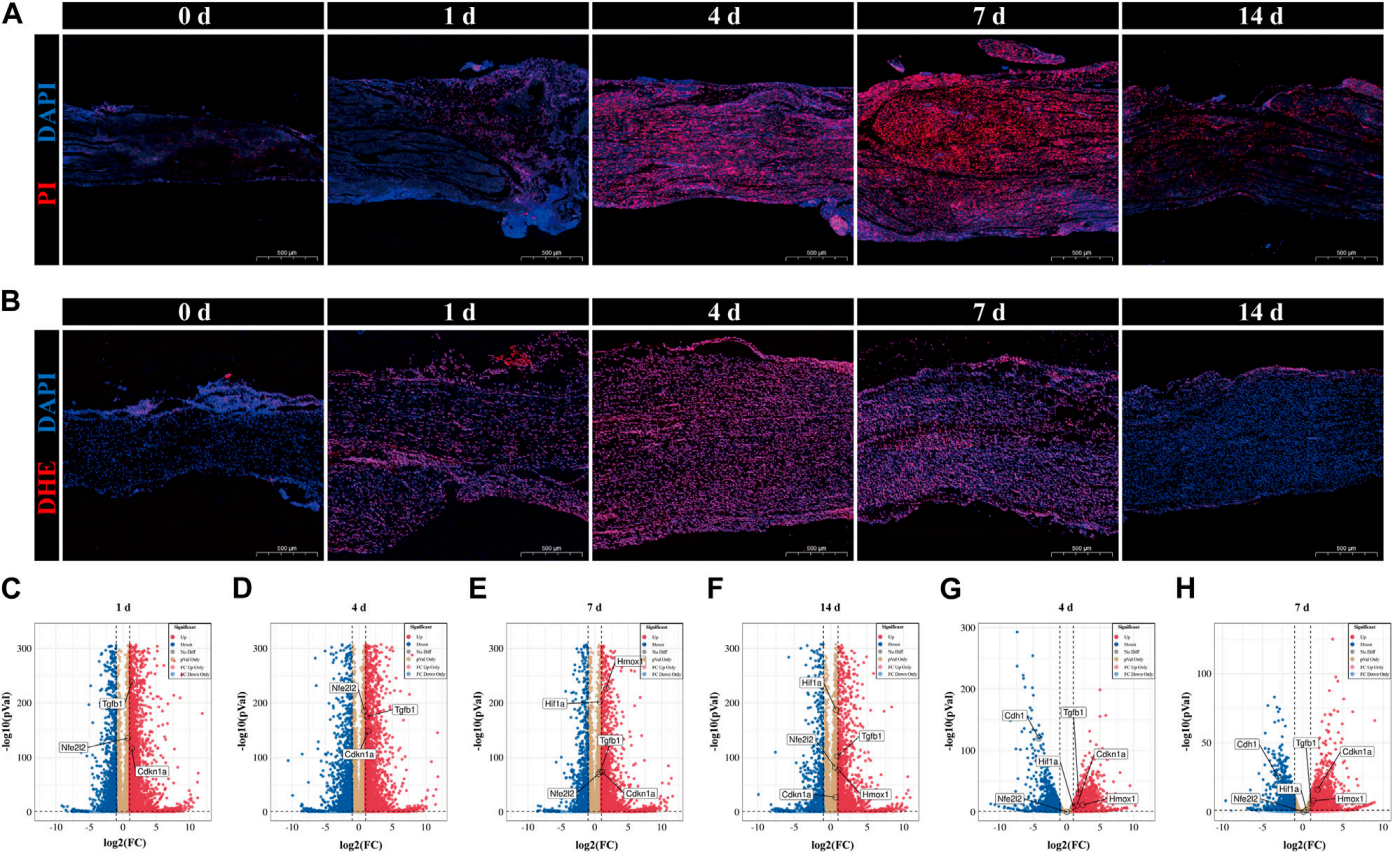
Validation of temporal alterations in ferroptosis and validation of datasets of hub genes. **(A)** Cell death in sciatic nerve was confirmed by PI and DAPI staining in the uninjured group (0 days) and 1-day group, 4-day group, 7-day group and 14-day group after nerve injury. PI in red and DAPI in blue. Scale bar = 500 μm. **(B)** ROS accumulation in sciatic nerve was authenticated by DHE staining in the 0-day (uninjured), 1-day, 4-day, 7-day and 14-day groups. DHE in red and DAPI in blue. Scale bar = 500 μm. **(C**-**F)** Volcano plots of DEGs of 1-day group, 4-day group, 7-day group, and 14-day group in sciatic nerve after crush injury compared to 0-day group (uninjured, control). The information came from SRP113121. **(G**, **H)** Volcano plots of DEGs of 4-day group, and 7-day group in sciatic nerve after crush injury compared to 0-day group (uninjured, control). The information came from GSE162548. **(C-H)** Red dots denote significantly upregulated genes and blue dots denote significantly downregulated genes with an adjusted *p*-value < 0.05 and |log2FC|>1.

Ferroptosis has been reported to be involved in pathophysiology after PNI (Gao et al., 2022); however, the trajectory of ferroptosis over time in this event remains unclear. Using PI and DAPI staining, we identified cell death in peripheral nerves after crush damage (Figure 6A). Cell death was uncommon in intact nerves and gradually increased 1 day after injury. The peak of cell death occurs between 4- and 7-day following damage, followed by a steady reduction. Next, we observed the temporal variations of ROS accumulation using DHE staining (Figure 6B). The results of ROS accumulation followed a similar pattern to that of PI staining; it started out gradually increasing, peaked 4–7 days after injury, and then started to decline. This implies that following peripheral nerve damage, ferroptosis initially developed and subsequently reduced, reaching its peak between 4 and 7 days.

Next, two questions demand our attention. Whether the chosen hub genes are expressed differently in rats after PNI, and whether there is a link between the temporal expression patterns of hub genes and the temporal progression of ferroptosis. The expression of hub genes was validated in other pertinent datasets with the same conditions, and the verification findings were shown as volcano plots. The 1-day group, 4-day group, 7-day group, and 14-day group results from data set SRP113121 are displayed in Figures 6C–F, respectively; the 4-day group and 7-day group results from data set GSE162548 are displayed in Figures 6G,H, respectively. In the first data set, the hub genes Nfe2l2, Tgfb1, and Cdkn1a showed substantial upregulation at all four time periods. But at the other hand, the upregulation of Hif1a and Hmox1 was only observed in the 7-day and 14-day groups. In the second data set, all six hub genes were substantially expressed except for Nfe2l2, which was not differently expressed in both time periods. Significant upregulation was seen for Tgfb1, Cdkn1a, Hif1a, and Hmox1, whereas Cdh1 was dramatically downregulated.

**FIGURE 7:**
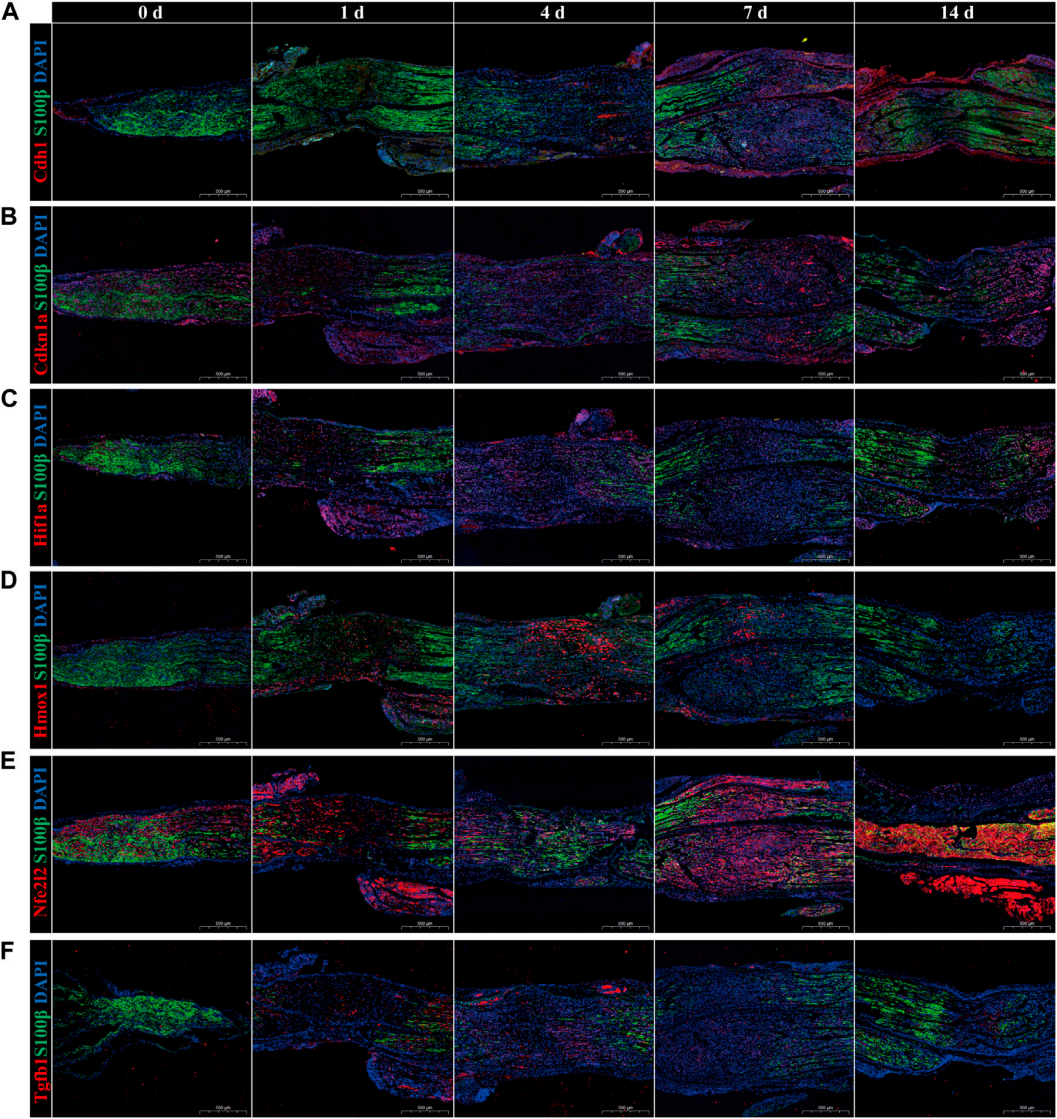
Immunofluorescence validation of temporal alterations in hub genes expression. The expression of hub genes **(A)** Cdh1, **(B)** Cdkn1a, **(C)** Hif1a, **(D)** Hmox1, **(E)** Nfe2l2, and **(F)** Tgfb1 in sciatic nerve in uncrushed (0 days) group, 1-day, 4-day, 7-day, and 14-day after crush injury. The hub gene was fluorescent in red, the Schwann cell marker S100β was fluorescent in green, and DAPI was fluorescent in blue. Scale bar = 500 μm. *p*-value < 0.05.

Subsequently, immunofluorescence was used to identify temporal changes in the expression of hub genes (Figures 7A–F). The red fluorescence corresponds to the hub genes, the green fluorescence to the Schwann cell marker S100β, and the blue fluorescence to DAPI. Results showed that in the uninjured sciatic nerve group (0 days), the Schwann cell marker S100β nearly covered the entire nerve. Upon inflicting a crush injury on the sciatic nerve, the total cell density at the crush sites decreased, and the fluorescence of S100 was attenuated or missing, indicating that the SCs at the crush sites were badly injured or had perished. In the sciatic nerves of the undamaged control group, the red fluorescence denoting the expression of Hmox1, Hif1a, and Tgfb1 was scarcely detectable. However, their expression steadily rose, reaching a peak between 4 and 7 days after injury, and then gradually reduced. While Cdh1, Cdkn1a, and Nfe2l2 were expressed at low levels in the intact sciatic nerves of the control group. The intensity of red fluorescence exhibited no significant change or even a tiny reduction after the injury, and after approximately 7 days, the intensity of red fluorescence progressively increased. Intriguingly, the expression variations of Hmox1, Hif1a, and Tgfb1 were consistent with the temporal alterations of ferroptosis after PNI, although the trends of Cdh1, Cdkn1a, and Nfe2l2 were opposite.

## Discussion

A peripheral nerve damage causes long-term suffering for patients and their families, as well as a significant societal burden. Although traditional therapeutic strategies for PNI, such as surgery, physical therapy, and pharmacological therapy, have improved significantly in recent years, the limitations of complete functional recovery continue to be major challenges (Modrak et al., 2020). Previous studies have shown that ferroptosis, a non-necrotizing form of programmed cell death, has been linked to the pathophysiology of neurodegenerative diseases like Alzheimer’s disease (Yan and Zhang, 2019), Huntington’s disease (Mi et al., 2019), and Parkinson’s disease (Mahoney-Sánchez et al., 2021), as well as acute central nervous system injuries (Shen et al., 2020) like stroke (Alim et al., 2019), traumatic brain injury (TBI) (Xie et al., 2019), and acute spinal cord injury (SCI) (Chen et al., 2020). Recently, it has been tentatively confirmed that ferroptosis happens following PNI (Guo et al., 2021; Gao et al., 2022). However, the specific biomarkers of ferroptosis after PNI remain unclear.

In this current study, we discovered and characterized six unique ferroptosis-associated hub genes as potential gene signatures and identified their temporal patterns after PNI. This study produced 52 DE-FRGs by performing a common intersection of DEGs from GSE177037 (3-day group, 5-day group, and 7-day group) and FRGs from FerrDb. GSEA was then carried out, and the outcomes demonstrated that mitochondrial-related gene sets were frequently enriched and upregulated, whereas lipid anabolic pathways were repeatedly enriched and downregulated. Next, a functional enrichment analysis of 52 DE-FRGs was conducted using the GO, KEGG, Reactome, and WikiPathways online databases, and the results suggested that Adipogenesis, the MAPK signaling pathway, the p53 signaling pathway, and the CD28 molecular family may be involved in ferroptosis after PNI. Cdkn1a, Cdh1, Hif1a, Hmox1, Nfe2l2, and Tgfb1 were subsequently evaluated as novel ferroptosis-related hub genes after PNI by comprehensive analysis in PPI network. The clustering correlation heatmap indicated that these six hub genes have a strong relation with mitochondria. Using immunofluorescence assay, Tgfb1, Hmox1 and Hif1a gene expressions increased after injury, peaked between 4 and 7 days, and then decreased gradually, which corresponded to the temporal variations in ferroptosis shown by PI and ROS staining. While Cdh1, Cdkn1a, and Nfe2l2 were diametrically opposed. Therefore, from the perspective of bioinformatics analysis, this work might serve as a useful reference for biomarkers of ferroptosis after PNI.

Based on our screening criteria of sensitivity, specificity, statistical significance, and originality in all nodes of the PPI network, six ferroptosis-associated gene signatures, including Cdh1, Cdkn1a, Nfe2l2, Tgfb1, Hmox1, and Hif1a was created.

CDH1, also referred to as E-cadherin, encodes a typical cadherin of the cadherin superfamily. Previous research has suggested that CDH1 may have anti-ferroptosis properties. *In vitro* and *in vivo*, ferroptosis susceptibility was raised by CDH1 silencing or ZEB1 overexpression, whereas susceptibility was lowered by CDH1 overexpression or ZEB1 silencing (Lee et al., 2020). And Neurofibromin (NF2) deficiency and low E-cadherin (CDH1) levels have been shown to increase ferroptosis vulnerability in meningiomas (Bao et al., 2021). The expression of CDKN1A (p21) is strictly regulated by the tumor suppressor protein p53, and this protein mediates the p53-dependent G1 phase arrest of the cell cycle in response to a range of stress stimuli. Some researchers have discovered that p53 could suppress ferroptosis by the induction of CDKN1A/p21 expression (Kang et al., 2019). Further research demonstrated that p21 levels are variably regulated in response to ferroptosis independent of p53 (Venkatesh et al., 2020). These evidences demonstrate that p21 tends to prevent ferroptosis. NFE2L2 encodes the transcription factor NRF2, which controls the expression of genes with antioxidant response elements (ARE) in their promoters, such as heme oxygenase 1 (HO-1) and so on (Kajarabille and Latunde-Dada, 2019). NRF2 plays a central role in protecting hepatocellular carcinoma cells against ferroptosis (Sun et al., 2016). Moreover, NRF2 expression and KEAP1 suppression improve cell proliferation and ferroptosis resistance (Fan et al., 2017). In PC12 cells, the expression of NRF2 increased iron storage capacity. While NRF2 knockdown made cells more susceptible to ferroptotic cell death (Liu et al., 2020). As a result, past research suggests that CDH1, CDKN1A, and NFE2L2 may play a major role in the ferroptosis defense system, which is compatible with the findings of our experimental verification.

HMOX1 encodes the heme-catabolic enzyme HO-1. As a downstream molecule of NRF2, HMOX1 promotes intracellular iron synthesis, which exerts an anti-ferroptotic effect. Ferroptosis caused by erastin is encouraged by HO-1 deficiency. The significant antiferroptotic function of HO-1 in renal epithelial cells may be a factor in the aggravation of AKI-related damage to proximal tubules (Adedoyin et al., 2018). Besides, WT mouse embryonic fibroblasts (MEFs) were more vulnerable to ferroptosis after HMOX1 knockdown (Nishizawa et al., 2020). As another downstream protein of NRF2, the transcription factor HIF1 acts as the principal regulator of cellular and systemic homeostasis in response to hypoxia. HIF1A could encode a subunit of HIF1. Inhibiting HIF-1 raises the free iron concentration, enhances mitochondrial iron buildup, and triggers ferroptosis under hypoxia (Ni et al., 2021). Destabilizing HIF1A accelerated tumor cell ferroptosis (Yang et al., 2019). These evidences suggest that HMOX1 and HIF1A inhibits the progression of ferroptosis. However, HMOX1 and HIF1A have been found to hasten ferroptosis in some specific circumstances. Prepubertal Di-(2-ethylhexyl) phthalate (DEHP) exposure caused ferroptosis in mice testes *via* the HIF1/HO-1 signaling pathway (Wu et al., 2022). Some claim that HO-1 is required for iron-dependent lipid peroxidation in ferroptosis and accelerates erastin-induced ferroptotic cell death (Kwon et al., 2015). HO-1 also handles BAY 11–7085-induced ferroptosis through the NRF2-SLC7A11-HO-1 pathway in response to the cellular redox status (Chang et al., 2018). TGFB1, a secreted ligand of the transforming growth factor-beta (TGF-β) superfamily, aids in the recruitment and activation of SMAD family transcription factors through binding to TGF-β receptors. TGFB1 is a double-edged sword in ferroptosis. In triple negative breast cancer (TNBC), hepatic leukemia factor (HLF) was regulated by TGFB1 secreted by tumor-associated macrophages. HLF transactivated GGT1 to promote ferroptosis resistance, driving TNBC cell proliferation, metastasis, and cisplatin resistance (Li et al., 2022). All this evidence implies that TGFB1 contributes to ferroptosis defense, but some studies demonstrate it can potentially induce ferroptosis. TGFB1 represses xCT expression *via* Smad3 activation and mildly increases lipid peroxidation, making HCC cells sensitive to GPX4 inhibition, which means TGFB1 raises ferroptosis risk (Kim et al., 2020).

The aforementioned researches indicate that some of these hub genes have the impact of reducing ferroptosis, whereas other genes may have the dual effect of promoting and inhibiting ferroptosis. Although it is unknown if TGFB1, HMOX1, and HIF1A have a significant role in promoting or inhibiting ferroptosis and how their dynamic changes occur, a thorough examination of their expression levels may suggest the presence of ferroptosis.

To further test our hypothesis, mitochondrial correlation analysis, dataset validation and experimental validation were performed. Mitochondria are the primary generators of ROS, the principal sites of iron consumption, and the master regulators of oxidative metabolism. Ferroptosis is significantly linked with mitochondrial morphological, bioenergetic, and metabolic damage (Battaglia et al., 2020). Through the clustering correlation heatmap of hub genes and DE-MRGs, Tgfb1, Hmox1, Hif1a, and Nfe2l2 were grouped into the same cluster, indicating their functional relevance. Moreover, these four ferroptosis-related hub genes were positively linked with the vast majority of DE-MRGs, indicating that they may be involved in the control of ROS production, iron consumption, and oxidative metabolism. Therefore, this study reflects the great association between hub genes and ferroptosis from the side, which is consistent with prior research findings (Fang et al., 2019; Li et al., 2019; Dong et al., 2021; Wu et al., 2022). According to the datasets’ validation findings, it can be shown that following PNI, the expression of Hif1a, Hmox1, and Tgfb1 is upregulated at all time nodes in the datasets SRP113121 and GSE162548, but Cdh1 is downregulated. Previous studies have shown that Cdh1 inhibits the development of ferroptosis. However, Hif1a, Hmox1, and Tgfb1 exert both positive regulatory and inhibiting effects on ferroptosis. Immunofluorescence assay revealed that following PNI, the expressions of Hif1a, Hmox1, and Tgfb1 gradually increased in the 1-and 3-day groups. The upregulated level steadily dropped after the expression level peaked at 4–7 days. This is in line with the overall trend of the dataset’s results. Additionally, it is hypothesized that Hif1a, Hmox1, and Tgfb1 are primarily responsible for encouraging ferroptosis following PNI. Non-etheless, the expression levels of Cdh1, Cdkn1a, and Nfe2l2 were initially downregulated or unaltered, followed by a general upregulation. Only Cdh1 validation results were consistent with immunofluorescence results among the datasets. Therefore, mitochondrial correlation analysis, dataset validation, and immunofluorescence assay confirmed that this ferroptosis-related gene signature composed of Hif1a, Hmox1, Tgfb1, Cdh1, Cdkn1a, and Nfe2l2 can roughly identify the presence of ferroptosis and temporal alterations following PNI.

In recent years, an increasing number of researchers have attempted to target ferroptosis treatment for acute CNS injures (Karuppagounder et al., 2018; Zhou et al., 2020; Abdul et al., 2021; Chen et al., 2021). The findings indicate that these therapies may have neuroprotective properties. Unfortunately, target ferroptosis therapy for acute PNI has received little research attention. This work enriches our knowledge of the pathophysiology of PNI. The specific biomarkers of ferroptosis after PNI were identified, which could assist in the rapid and reliable test of ferroptosis and provide potential therapeutic targets for ferroptosis after PNI as well as the therapeutic window for guiding treatment. However, the scope of this investigation remains limited. In the future, molecular biology experiments and clinical trials of drugs and small compounds targeting hub genes need to be conducted and eventually alleviate the burden on patients with PNI.

## Data Availability

The datasets presented in this study can be found in online repositories. The names of the repository/repositories and accession number(s) can be found in the article/supplementary material.
